# Endovascular thrombectomy for acute ischemic stroke in elderly patients with atrial fibrillation

**DOI:** 10.1186/s12883-022-02631-3

**Published:** 2022-03-17

**Authors:** Jincheng Jiao, Sheng Liu, Chang Cui, Yuezhou Cao, Zhenyu Jia, Hailei Liu, Chendong Wang, Yu Hang, Heng Ni, Minglong Chen, Mingfang Li, Haibin Shi

**Affiliations:** 1 Division of Cardiology, The First Affiliated Hospital of Nanjing Medical University, 300 Guangzhou Road, Nanjing, 210029 China; 2 Division of Interventional Radiology, The First Affiliated Hospital of Nanjing Medical University, Nanjing, China

**Keywords:** Acute ischemic stroke, Atrial fibrillation, Elderly, Endovascular thrombectomy

## Abstract

**Background:**

To assess the clinical outcomes after endovascular thrombectomy (EVT) in elderly large vessel occlusion (LVO)-related acute ischemic stroke (AIS) patients with atrial fibrillation (AF).

**Methods:**

Between January 2019 and December 2020, consecutive AF patients who received EVT due to anterior-circulation stroke were enrolled. The primary outcome was modified Rankin scale (mRS) score at 90 days. Secondary outcomes included all-cause mortality, the recanalization status after EVT (assessed using modified thrombolysis in cerebral infarction scale, mTICI) and any intracranial hemorrhage (ICH). A multivariate logistic regression model was performed to identify predictors of the functional outcome.

**Results:**

A total of 148 eligible patients were finally enrolled. Among them, 42 were ≥ 80 years old. Compared to their younger counterparts, patients aged ≥80 years had lower likelihood of good functional outcome (mRS score 0–2) at 90 days (26.2% vs. 48.1%, *P* = 0.015), less satisfied recanalization (mTICI, 2b-3) (78.6% vs. 94.3%, *P* = 0.004) and higher all-cause mortality rate (35.7% vs. 14.2%, *P* = 0.003). A multivariable logistic regression analysis showed that age ≥ 80 years at baseline were the significant predictors for a poor functional outcome (OR: 3.72, 95% CI: 1.17–11.89, *p* = 0.027). Intravenous thrombolysis (IVT) prior to EVT and longer time intervals from onset of symptoms to EVT tended to be associated with poor functional outcome in patients ≥80 years old.

**Conclusions:**

Age ≥ 80 years was a significant predictor of unfavorable outcomes after EVT for AIS patients with AF. An increased risk of adverse events must be balanced against the benefit from EVT in elderly patients with AF.

## Background

Atrial fibrillation (AF) is the most common cause of cardioembolism, accounting for about 20–30% of acute ischemic stroke (AIS) [1]. Approximately one-third of AISs occur in patients aged ≥80 years [2]. Considering that the incidence of AF and the risk of AF-related stroke increases significantly with age, there is a high proportion of elderly AIS patients with AF, especially in patients aged ≥80 years [3].

Since the publication of five major randomized controlled trials (RCTs) [4], endovascular thrombectomy (EVT) has been recommended as a standard treatment applied within 6 h of an AIS in the anterior circulation due to large vessel occlusion (LVO), even in very elderly patients (≥80 years old). Subsequently, for patients within 6–24 window hours who meet the DAWN [5] or DEFUSE 3 [6] eligibility criteria, EVT was also recommended. However, the meta-analysis of the five RCTs showed that at 90 days after AIS, the modified Rankin scale (mRS) scores was significantly higher (lower score indicating better outcome) in the patients aged ≥80 years compared with those at younger ages [7]. Recently, in a ‘real world’ study, EVT carried a higher risk of hemorrhagic complications than medical treatment in elderly patients [8].

Due to their multi-morbidities, declining multiple organ function, poor tolerance to invasive or noninvasive treatment, possible prior use of anticoagulants and high risk of bleeding after intravenous thrombolysis (IVT), elderly AIS patients with AF practically have limited therapeutic options and poor outcomes. In the meantime, the benefit of EVT for LVO-related AIS patients with AF who are ≥80 years was less clearly elucidated.

This study aims to assess the clinical outcomes after EVT in elderly LVO-related AIS patients with AF and their association with previous medical histories and preoperatively available clinical variables.

## Methods

### Study design and study population

This is a single-center retrospective observational study. From January 2019 to December 2020, consecutive patients with AIS who received EVT were enrolled from the stroke center in this study. All patients suspected to have AIS received post-processed computed tomography angiography (CTA) scan to confirm the occlusion situation of blood vessel in head and neck. Since September 2019, CTA has been replaced by computed tomography perfusion (CTP). The treatment strategies were developed by experienced neurologists and interventional neuro-radiologists after the evaluation of patients and images according to the American Heart Association/American Stroke Association (AHA/ASA) guidelines [9]. In particular, the indication for EVT in this study were as follows: (1) pre-stroke mRS score < 2; (2) AIS due to LVO in the anterior circulation confirmed by CTA or CTP; (3) ≥18 years old; (4) NIHSS score of ≥6; (5) ASPECTS of ≥6; and (6) patients within 6 h of symptom onset or within 6 to 24 h of last known normal who meet the DAWN or DEFUSE 3 eligibility criteria [5, 6, 9].

The exclusion criteria in this study were as follows: (a) patients without AF; (b) patients with in-hospital stroke; (c) loss to follow up or lack of baseline characteristics; (d) patients who only received angiography or intra-arterial thrombolysis; (e) patients with acute vertebrobasilar occlusion. The study was approved by the medical ethics committee of the local university and there is no conflict of interest among all authors.

### Endovascular thrombectomy

The procedure detail has been described in our previous report [10]. In brief, local anesthesia supplemented by conscious sedation was performed before the procedure in all patients. A Solitaire AB device (Medtronic, Irvine, California, USA) was used during EVT, combined with aspiration through the corresponding guiding catheter (Envoy, Cordis) or distal access catheter (Navien, ev3). Blood flow recovery was evaluated after each EVT. For the residual stenosis in cases with in situ thrombosis, whether to perform balloon angioplasty and stent placement was at the discretion of the operator. Intra-arterial thrombolysis, or intra-catheter tirofiban administration might be considered as rescue therapies.

### Baseline and clinical assessment

The data collected were age, gender, previous medical history including AF, hypertension, diabetes mellitus, coronary atherosclerosis disease (CAD), heart failure, previous stroke or transient ischemic attack (TIA), and valvular heart disease, and previously used antithrombotic drugs. The National Institutes of Health Stroke Scale (NIHSS) score, Alberta Stroke Program Early CT Score (ASPECTS) on admission and CHA_2_DS_2_-VASc score (variables age, heart failure, hypertension, diabetes mellitus, vascular disease, stroke and systemic embolism, gender) was assessed right after patients arrived in the hospital. Process time including stroke symptom onset to door, door to puncture, and puncture to reperfusion was recorded. Peripheral blood of each patient was collected for analysis of complete blood count, prothrombin time (PT), activated partial thromboplastin time (APTT), international normalized ratio (INR), fibrin, and D-dimer, cardiac troponin T (c-TnT), serum electrolyte, liver and renal function. Patients received scheduled follow-up visits by neurologists or by phone call interview at 90 days post-stroke.

### AF diagnosis and effective anticoagulation definition

In this study, AF were diagnosed based on the previous history of AF or electrocardiographic documentation of AF episode on admission. In addition, one or more 24- to 72-h continuous ECG patch monitoring was conducted in patients with no evidence of AF and no obvious stenosis after recanalization of the occluded vessel, which was defined as absence of intracranial atherosclerosis causing ≥50% luminal stenosis in arteries supplying the area of ischemia. Non-paroxysmal AF was defined as permanent or persistent AF. Patients on warfarin with an INR ≥1.7 or last novel oral anticoagulant (NOAC) intake < 48 h before onset of stroke was considered to be effectively anticoagulated [11].

### Time to (re)start oral anticoagulants after the procedure of endovascular thrombectomy

When to initiate oral anticoagulants (OACs) was at the discretion of treating doctors. Patient’s age, the severity of postoperative symptoms, the size of cerebral infarction area and the occurrence of hemorrhagic transformation were the most important factors to consider.

### Clinical outcomes

The primary outcome was defined as the mRS score at 90 days. The secondary outcome was all-cause mortality at 90 days, the recanalization status after EVT (assessed using modified thrombolysis in cerebral infarction scale, mTICI) and any intracranial hemorrhage (ICH), which included hemorrhagic transformation (HT) [12, 13].

### Statistical analysis

Statistical Package for the Social Sciences (SPSS) software 26.0 (IBM, Armonk, NY) was used for all the statistical analyses. Continuous variables were presented as mean ± standard deviation (SD) or median with the interquartile range (IQR). To compare the difference between the patients aged ≥80 years and those aged < 80 years, unpaired Student’s *t-*test was used if continuous variables were normally distributed, or nonparametric test was used if not normally distributed. Categorical variables were expressed as counts with percentages and compared using Chi-square tests or Fisher’s exact tests. Significance was defined when *P* < 0.05. Multivariate logistic regression was performed to determine whether age ≥ 80 years and other baseline characteristics had an independent impact on the prognosis of EVT. Variables with a *P* value < 0.05 in univariate analysis were included in the multivariate logistic regression model. The odds ratios (ORs) and corresponding 95% confidence intervals (Cis) were calculated to assess the association.

## Results

### Baseline characteristics

**Fig. 1:**
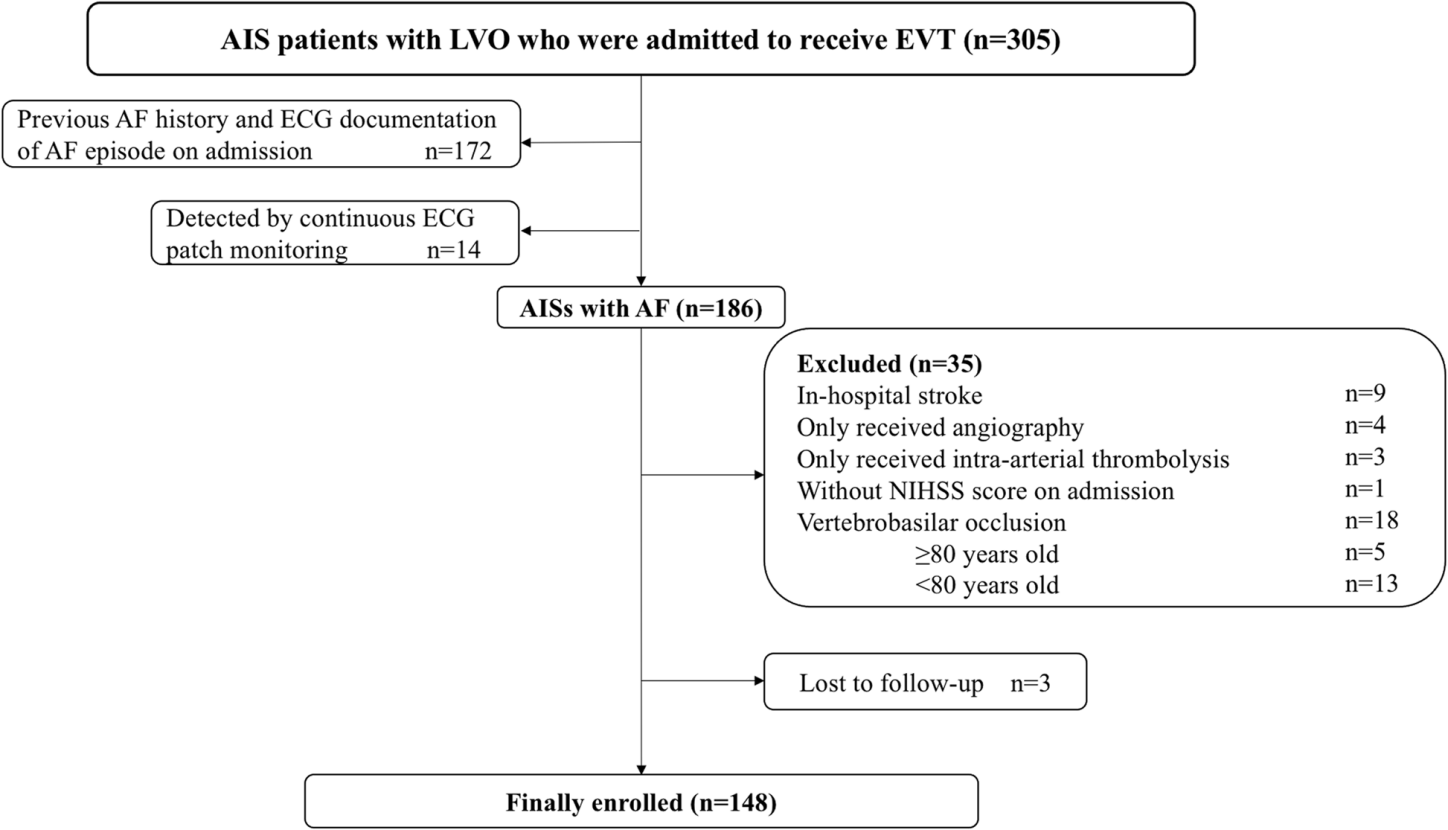
Flow chart of patients included in the present study. AF = atrial fibrillation; AIS = acute ischemic stroke; LVO = large intracranial vessel occlusion; EVT = endovascular thrombectomy; ECG = electrocardiogram; NIHSS = National Institute of Health Stroke Scale

During the study period, a total of 305 AIS patients received EVT. The final analysis included 148 patients with AF who underwent EVT (Fig. 1). There were 14 patients newly detected AF by continuous ECG patch monitoring. Among them, 42 patients (28.4%) were ≥ 80 years old and the remaining 106 patients (71.6%) were <  80 years. Compared to those at younger age, patients aged ≥80 years had lower level of eGFR (73.9 ± 17.2 ml/min vs. 88.4 ± 18.0 ml/min, *P* < 0.001), less prior IVT (21.4% vs. 41.5%, *P =* 0.022) and higher CHA_2_DS_2_-VASc score (5 [IQR, 4–5] vs. 3 [IQR, 2–4], *P* < 0.001). There were no significant differences in other baseline clinical characteristics between these two groups (Table 1). The rate of previous anticoagulant use was 7.4% (11/148) in all the enrolled patients, and 2.4% (1/42) in those ≥80 years.

**Table 1:** Baseline clinical characteristics

Characteristic	Total (*n* = 148)	< 80 y (*n* = 106)	≥80 y (*n* = 42)	*P* value
**Men, n (%)**	60 (40.5)	46 (43.4)	14 (33.3)	0.261
**Age (y)**	73.3 ± 10.2	68.9 ± 8.6	84.3 ± 2.9	**< 0.001**
**Type of AF, n (%)**				
Non-paroxysmal AF	106 (71.6)	77 (72.6)	29 (69.0)	0.662
**Pre-stroke mRS**				0.510
0	136 (91.9)	96 (90.6)	40 (95.2)	
1	12 (8.1)	10 (9.4)	2 (4.8)	
**Comorbidities, n (%)**				
Heart failure	6 (4.1)	3 (2.8)	3 (7.1)	0.352
Hypertension	106 (71.6)	74 (69.8)	32 (76.2)	0.438
Diabetes	34 (23.0)	25 (23.6)	9 (21.4)	0.779
Pre-stroke/TIA	35 (23.6)	22 (20.8)	13 (30.1)	0.188
Coronary artery disease	17 (11.5)	10 (9.4)	7 (16.7)	0.213
**Valvular heart disease, n (%)**				
Rheumatic valvular heart disease	11 (7.4)	10 (9.4)	1 (2.4)	0.181
Cardiac valve replacement	5 (3.4)	5 (4.7)	0 (0)	0.322
**Antithrombotic, n (%)**				
Anticoagulation	11 (7.4)	10 (9.4)	1 (2.4)	0.181
warfarin	4 (2.7)	4 (3.8)	0 (0)	1.000
NOAC	7 (4.7)	6 (5.6)	1 (2.4)	1.000
Antiplatelet	31 (20.9)	20 (18.9)	11 (26.2)	0.324
**Wake-up stroke, n (%)**	27 (18.2)	20 (18.9)	7 (16.7)	0.755
**Clinical scores, median (IQR)**				
Baseline NIHSS score	16 (12–21)	15(12–21)	18 (12–22)	0.189
CHA_2_DS_2_-VASc score	4 (2–5)	3(2–4)	5 (4–5)	**< 0.001**
**Serological indicator, mean ± SD**				
Cardiac troponin T, ng/L	20.3 ± 21.7	19.8 ± 23.3	21.3 ± 17.7	0.726
D-dimer, mg/L	2.4 ± 4.4	2.6 ± 5.0	1.7 ± 1.6	0.113
INR	1.2 ± 0.5	1.2 ± 0.6	1.1 ± 0.1	0.408
Alanine aminotransferase, U/L	33.2 ± 15.4	34.2 ± 15.8	30.7 ± 14.6	0.212
Aspartate transaminase, U/L	33.2 ± 12.4	33.2 ± 12.3	33.3 ± 13.2	0.956
eGFR, ml/min	84.3 ± 18.8	88.4 ± 18.0	73.9 ± 17.2	**< 0.001**
White Blood Cell, 10^9/L	8.6 ± 3.0	8.8 ± 3.0	8.2 ± 2.8	0.268
Platelet, 10^9/L	173.8 ± 56.2	175.7 ± 57.6	169.0 ± 53.8	0.518
Neutrophil-to-lymphocyte ratio	6.4 ± 4.1	6.4 ± 4.0	6.2 ± 4.6	0.800
**ASPECTS**	7 (6–9)	7 (6–9)	7 (6–9)	0.482
**Occlusion site, n (%)**				
Middle cerebral artery	100 (67.6)	73 (68.9)	27 (64.3)	0.591
M1	80 (54.1)	60 (56.6)	20 (47.6)	0.368
M2	20 (13.5)	13 (12.3)	7 (16.7)	0.368
Internal carotid artery	48 (32.4)	33 (31.1)	15 (35.7)	0.591
**Prior IVT, n (%)**	53 (35.8)	44 (41.5)	9 (21.4)	**0.022**
**TOAST, n (%)**				
Cardio-embolic	136 (91.9)	99 (93.4)	37 (88.1)	0.516
Large artery atherosclerosis	12 (8.1)	7 (6.6)	5 (11.9)	0.516

*AF* atrial fibrillation, *mRS* modified Rankin Scale, *TIA* transient ischemic attack, *DOAC* direct oral anticoagulants, *NIHSS* National Institute of Health Stroke Scale, *CHA*_*2*_*DS*_*2*_*-VASc* congestive heart failure, hypertension, age ≥ 75 years (doubled), diabetes, stroke (doubled), vascular disease, age 65–74 years, and sex category (female), *IVT* intravenous thrombolysis, *eGFR* estimated glomerular filtration rate, *ASPECTS* Alberta Stroke Program Early CT Score, *INR* international normalized ratio, *IQR* interquartile range, *SD* standard deviation

eGFR = 141 × min (Scr/ĸ, 1)α × max (Scr/ĸ, 1) − 1.209 × 0.993Age × 1.018 [if female] × 1.159 [if black], where Scr is serum creatinine, ĸ is 0.7 for females and 0.9 for males, α is −0.329 for females and − 0.411 for males, min indicates the minimum of Scr/ĸ or 1, and max indicates the maximum of Scr/ĸ or 1

### Operation parameters

The median time from puncture to reperfusion in patients ≥80 years was significantly longer than in those at younger age (70 min [IQR, 50–124 min] vs. 54 min [IQR, 40–84 min], *P* = 0.011). There were no significant differences in other time intervals between the two groups, including onset to door time interval, door to puncture time interval and total procedure duration. Patients aged ≥80 years tended to have more retrieval attempts (2 [IQR, 1–2] vs. 1 [IQR, 1–2], *P* = 0.064) than those aged < 80 years. Detailed procedural parameters are shown in Table 2.

**Table 2:** Operation parameters

Characteristic	Total (*n* = 148)	< 80 y (*n* = 106)	≥80 y (*n* = 42)	*P* value
**Time intervals (min), median (IQR)**				
Onset to door	195 (139–278)	198 (152–264)	179 (106–315)	0.550
Door to puncture	76 (64–98)	75 (64–97)	76 (64–99)	0.467
Puncture to reperfusion (***n*** **= 140**)	60 (44–96)	54 (40–84)	70 (50–124)	**0.011**
Total procedure	341 (293–455)	341 (294–442)	343 (283–477)	0.798
**Procedural features, n (%)**				
Retrieval attempts, median (IQR)	1 (1–2)	1 (1–2)	2 (1–2)	0.064
Solitaire	116 (78.4)	82 (77.4)	34 (80.9)	0.632
Combined with Intra-arterial thrombolysis	5 (3.4)	4 (3.7)	1 (2.4)	1.000
Balloon dilatation	7 (4.7)	5 (4.7)	2 (4.7)	1.000

*IQR* interquartile range

### Clinical outcomes

During follow-up, 71 (60.2%) patients out of the 118 survivors were prescribed with OACs after EVT. Warfarin was used in 17 patients, rivaroxaban in 18 patients, and dabigatran in 36 patients. Overall, 30 patients (re)started OACs within 14 days after EVT, and another 41 patients received OACs 14 days after EVT.

**Fig. 2:**
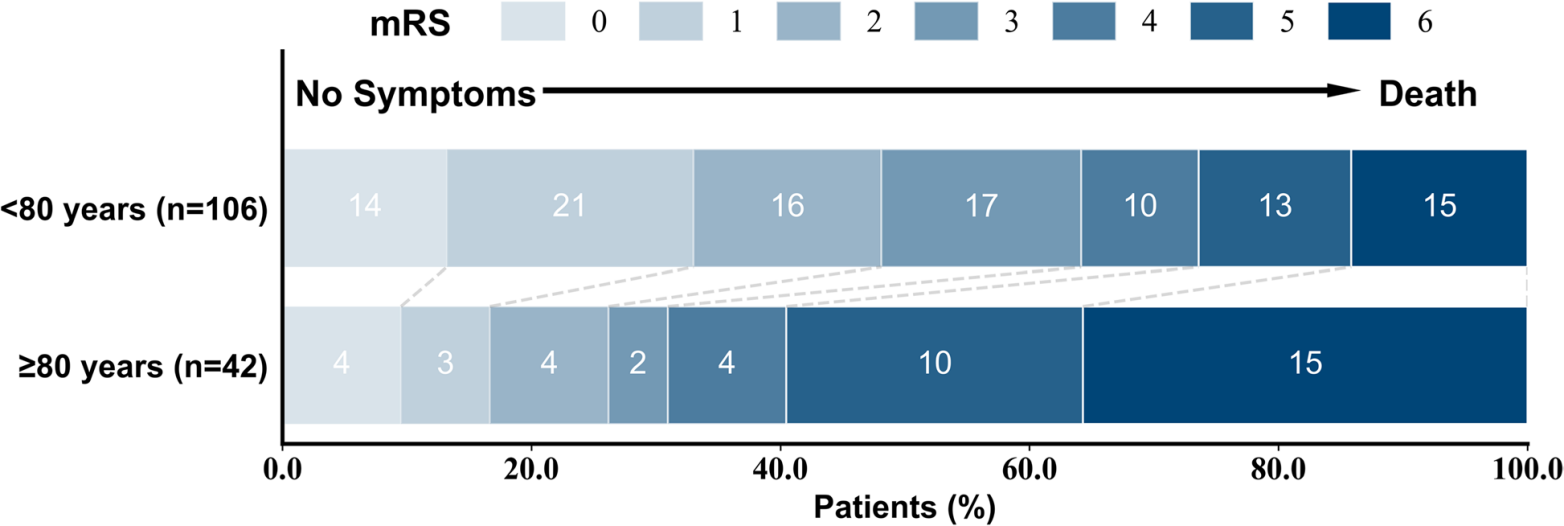
Functional outcome on the modified Rankin scale (mRS) at 90 days in patients aged ≥80 years vs. < 80 years

Patients aged ≥80 years undergoing EVT had a lower rate of favorable functional outcome with mRS sore 0–2, compared to those at younger age (26.2% vs. 48.1%, *P* = 0.015) as shown in Table 3 and Fig. 2. In the multivariate logistic regression model, age ≥ 80 years old (OR: 3.72, 95% CI: 1.17–11.89, *P* = 0.027), non-paroxysmal AF (OR: 3.74, 95% CI: 1.23–11.38, *P* = 0.020), higher baseline NIHSS score (OR: 1.17, 95% CI: 1.08–1.27, *P* < 0.001) and higher c-TnT level (OR: 1.04, 95% CI: 1.01–1.09, *P* = 0.039) significantly predicted a poor functional outcome of mRS score 3–6 after adjustment (Table 4).

**Table 3:** Clinical outcomes

Outcome	Total (*n* = 148)	< 80 y (*n* = 106)	≥80 y (*n* = 42)	*P* value
**Primary endpoint, n (%)**				
mRS score 0–2 at 90 days	62 (41.9)	51 (48.1)	11 (26.2)	**0.015**
**Secondary endpoint, n (%)**				
Mortality at 90 days	30 (20.2)	15 (14.2)	15 (35.7)	**0.003**
mTICI 2b-3	133 (89.9)	100 (94.3)	33 (78.6)	**0.004**
Intracranial hemorrhage	48 (32.4)	34 (32.1)	14 (33.3)	0.883

*mRS* modified Rankin Scale, *mTICI* modified thrombolysis in cerebral infarction

**Table 4:** Univariate and multivariate logistic regression to predict unfavorable functional outcome (a Modified Rankin Scale score of 3–6) at 90 days

	Univariate		Multivariate model	
	OR (95% CI)	*P* value	OR (95% CI)	*P* value
Age ≥ 80 years	2.61 (1.19–5.74)	0.015	3.72(1.17–11.89)	0.027
Non-paroxysmal AF	2.39 (1.15–4.94)	0.019	3.74(1.23–11.38)	0.020
CHA_2_DS_2_-VASc score	1.48 (1.18–1.85)	< 0.001		
Baseline NIHSS score	1.19 (1.11–1.28)	< 0.001	1.17(1.08–1.27)	< 0.001
Antiplatelet	3.04 (1.22–7.61)	0.017		
c-TnT	1.05 (1.01–1.09)	0.008	1.04(1.01–1.09)	0.039
eGFR	0.98 (0.96–0.99)	0.013		
D-dimer	1.42 (1.08–1.86)	0.011		
Neutrophil-to-lymphocyte ratio	1.14 (1.04–1.25)	0.006		

Abbreviations as in Table 1

Sex, heart failure, hypertension, diabetes, pre-stroke/TIA, CAD, valvular heart disease, anticoagulation, antiplatelet, wake-up stroke, pre-stroke mRS, time to (re)started OACs, ALT, AST, white blood cell, platelet and several time intervals from onset of symptoms to treatment showed in Table 2 were also conducted univariate analysis and no significant differences were found

In total, 30 patients died during 90 days after EVT. The cause of death was massive cerebral infarction in 11 patients, fatal ICH in 12 patients, serious lung infection in 6 patients and fatal heart failure in 1 patient. Patients aged ≥80 years had a higher all-cause mortality rate compared to those at youngers (35.7% vs. 14.2%, *P* = 0.003). The rate of good revascularization in patients aged ≥80 years was significantly lower than that in those at younger age (mTICI 2b-3, 78.6% vs. 94.3%, *P* = 0.004). However, there was no remarkable difference in the proportion of ICH between these two groups (33.3% vs. 32.1%, respectively, *P* = 0.883) as shown in Table 3. Actually, 10.8% patients had symptomatic ICH in this study. However, the accurate proportion of asymptomatic intracranial hemorrhage was not clear, since not every patient returned to hospital for CT or MRI scan. What’s more, no serious adverse events (neurological deterioration, vascular events, or death) occurred in patients receiving OACs during the 90-day follow-up.

In a univariate analysis of patients aged ≥80 years, IVT prior to EVT tended to be associated with worse functional outcomes (OR: 3.48, 95% CI: 0.38–31.63). Shorter time intervals from onset of symptoms to treatment were numerically associated with better functional outcome (Table 5).

**Table 5:** Univariate analysis of the association of unfavorable functional outcome (a Modified Rankin Scale score of 3-6) at 90 days with procedural parameters in patients aged ≥80 years

	Univariate	
	OR (95% CI)	*P* value
Intravenous rt-PA use	3.48 (0.38–31.63)	0.268
**Time intervals (per hour)**		
Onset to door	1.13 (0.83–1.53)	0.442
Onset to puncture	1.14 (0.85–1.53)	0.378
Puncture to reperfusion	1.52 (0.65–3.56)	0.337
Total procedure	1.16 (0.88–1.54)	0.298

*rt-PA* recombinant tissue-Plasminogen Activator, alteplase

## Discussion

In this retrospective observational study, we found that age ≥ 80 years was an independent predictor for poor functional outcome at 90 days after EVT in LVO-related AIS patients with AF. In addition, older age might lead to unfavorable recanalization rates and higher all-cause mortality rate. This suggested that the optimization of selection criteria for such elderly patients with AF to undergo EVT was urgently needed to improve their prognosis.

Several single-center studies had shown that patients > 80 years old had worse mRS outcome (90-day mRS 3–6) and higher overall mortality [8, 14]. According to the recent DAWN study and MR CLEAN study, EVT was effective in patients > 80 years old [5, 15]. However, only 25 patients aged ≥80 years who had an NIHSS score of 10 or higher and < 21 mL of infarct volume on imaging were included in DAWN study. A ‘real world’ study found that baseline high NHISS score and the incidence of hemorrhage were the two independent predictors of poor outcome in the elderly patients [8]. However, to the best of our knowledge, the association of age with the prognosis of EVT in patients with AF was less clearly elucidated.

Patients with AF-related stroke always have a heavy thrombus burden, which can easily lead to the occlusion of large intracranial vessels and massive cerebral infarction [16]. AF has been confirmed as an independent predictor for a poor outcome for AIS patients [17]. AIS patients with AF were often at high risk of ICH after IVT, which is associated with larger territories of hypoperfusion and larger infarct volumes [18]. Although EVT is recommended for selected AIS patients, both the subgroup analysis of the MR CLEAN study [15] and a single-center observational study [19] found that compared with those without AF, AIS patients with AF receiving thrombectomy tended to have poor mRS scores at 90 days. In addition, increased risk of ICH after EVT in AIS patients with AF was also found in another study [20].

Elderly patients with AF have poorer blood vessel quality, which may lead to unsuccessful recanalization and ICH. The proportion of ICH after EVT in this study was about 32%, which was similar to the findings in the DIRECT-MT study (33.3%) and subgroup analysis of the BEST study (43.9% in AF group, 27% in non-AF group) [12, 20]. The ENDOSTROKE study emphasized that older age was related to a decrease in clinically successful recanalization in anterior circulation, particularly if over 80 years [21]. Shear force due to abnormal blood flow caused by persistent or permanent AF can damage the cerebrovascular endothelium and promote the formation of artery plaques, atherosclerosis, and even stenosis [22], which may increase the difficulty of recanalization and risk of ICH.

To explore whether the procedure-related parameters have an impact on 90-day prognosis in elderly patients with AF, we performed a subgroup analysis of 42 elderly patients. The time intervals were not meaningful predictors in the univariate analysis, which may be due to the small sample size of the present study. However, reducing time intervals in total procedure tended to improve the functional outcome. Compared to those with stroke due to cervical carotid atherosclerosis, patients with AF-related stroke have less extensive collateral circulation. Previous studies have shown that the degree of intracranial vascular stenosis is related to the establishment of collateral circulation, and severe vascular stenosis can promote extensive collateral circulation [23]. AF-related stroke occurs when a cardiogenic thrombus breaks off. Therefore, collateral circulation fails to establish timely in this circumstance. From a practical perspective, the data from the present study suggest that earlier recanalization is important in elderly patients.

The present study also showed that compared with direct thrombectomy, bridging therapy with intravenous alteplase use might increase the risk of poor functional outcome. As a matter of fact, the DIRECT-MT study have demonstrated that with regard to functional outcome, EVT alone was noninferior to EVT combined with prior intravenous alteplase administered within 4.5 h after symptom onset in acute ischemic stroke from LVO [12]. Therefore, direct thrombectomy may have more beneficial effect on functional outcome in elderly AIS patients with AF.

In addition to older age, the present study suggests that non-paroxysmal AF, higher baseline NIHSS score and higher level of baseline c-TnT was associated with poor clinical outcomes at 90 days after EVT in elderly AIS patients with AF. These variables have been identified as the risk factors predicting the poor outcomes after stroke in prior studies [7, 24–27]. In elderly patients with these risk factors, the benefit and risk of EVT needs to be taken into consideration before the decision to perform the procedure is made.

The use of anticoagulants can effectively prevent stroke in elderly patients with AF [11]. In the present study, few elderly people with AF were effectively anticoagulated before the onset of AIS. Considering the poor prognosis of thrombectomy in elderly AF patients, it is urgent to emphasis the importance of primary thromboprophylaxis using anticoagulants in this population.

This study has some limitations. First, it was a single-center retrospective analysis with small sample size. Second, only available preoperative and interventional data were analyzed, while many other variables including cardiac structural and functional parameters measured using 2-dimensional echocardiography, level of B-type natriuretic peptide (BNP) and some other serological indicators were not assessed. Therefore, further larger-scale studies are needed to clarify the treatment effect of EVT in elderly patients with AF.

## Conclusions

Age ≥ 80 years was a significant predictor of unfavorable outcomes after EVT for AIS patients with AF. An increased risk of adverse events must be balanced against the benefit from EVT in elderly patients with AF. More efforts are needed to improve the use of anticoagulation in elderly AF patients for stroke prevention.

## Data Availability

The datasets used and/or analyzed during the current investigation are available upon reasonable request from the corresponding author.

## Abbreviations

EVT: Endovascular thrombectomy
AF: Atrial fibrillation
LVO: Large vessel occlusion
IVT: Intravenous thrombolysis
mTICI: modified thrombolysis in cerebral infarction scale
mRS: Modified Rankin Scale
ICH: Intracranial hemorrhage
NIHSS: National Institutes of Health Stroke Scale
NOAC: Novel oral anticoagulant
